# Case Report: Early detection and intervention of congenital portosystemic shunts in children

**DOI:** 10.3389/fonc.2023.1027238

**Published:** 2023-05-05

**Authors:** Ying Zhang, Tianzhuo Yu, Yanhong Mi, Wenzhi Zhang, Gaoyi Yang

**Affiliations:** ^1^ Department of Ultrasound, Hangzhou Red Cross Hospital and Affiliated Hangzhou Chest Hospital, Zhejiang University School of Medicine, Hangzhou, China; ^2^ Department of Radiology, The Children’s Hospital, Zhejiang University School of Medicine, National Clinical Research Center for Child Health, Hangzhou, China

**Keywords:** child, congenital portosystemic shunts, color Doppler ultrasound, diagnosis, hepatic tumor

## Abstract

Congenital portosystemic shunts (CPSS) are rare vascular anomalies that cause abnormal communications between the portal and systemic venous systems and may be incidentally detected on imaging or *via* abnormal laboratory parameters due to the lack of specificity in the condition’s clinical presentation. Ultrasound (US) is a common tool for examining abdominal solid organs and vessels and is the initial imaging modality for diagnosing CPSS. Here we report the case of an 8-year-old Chinese boy with CPSS diagnosed using color Doppler US. Doppler US first found intrahepatic tumor, then revealed that the left portal vein was directly communicating with the inferior vena cava, and the boy was finally diagnosed with intrahepatic portosystemic shunts. Interventional therapy was employed to occlude the shunt. During the follow-up, the intrahepatic tumor disappeared and no complications. Hence, to be able to differentiate such vascular anomalies, clinicians should be fairly acquainted with the normal ultrasonographic anatomical features in daily clinical work. Furthermore, increased disease awareness and advances in imaging equipment and technology are essential for CPSS diagnosis.

## 1 Introduction

Congenital portosystemic shunts (CPSS) are an abnormal connection between portal veins (PV) and systemic veins, resulting in varying degrees of portal blood flow diversion directed away from the liver to the systemic circulation. It may occur due to incomplete vascular remodeling between the embryonic and fetal hepatic and perihepatic circulations during fetal development (1, 2). The prevalence in neonates is approximately 1 in 30,000–50,000 (3). CPSS may present with various symptoms, including hepatic multiple tumors, galactosemia, neonatal cholestasis, abnormal liver function, hepatic encephalopathy (HE), among others (3, 4).

Color Doppler ultrasound (US) is the first imaging modality of choice for detecting CPSS and can demonstrate PV waveform, vascular shunts, thrombosis as well as intrahepatic nodules and other related complications (2, 5). Early diagnosis using Doppler US allows the prompt management of potentially life-threatening manifestations, ultimately improving the outcome in these patients (3). This report aimed to describe the case of a patient with CPSS with multiple hepatic tumors diagnosed using color Doppler US in Hangzhou Red Cross Hospital as well as the clinical progress from initial diagnosis to treatment improvement.

## 2 Case description

An 8-year-old Chinese boy visited our hospital and presented with emaciation without symptoms. He was previously healthy without known medical conditions.

**Figure 1:**
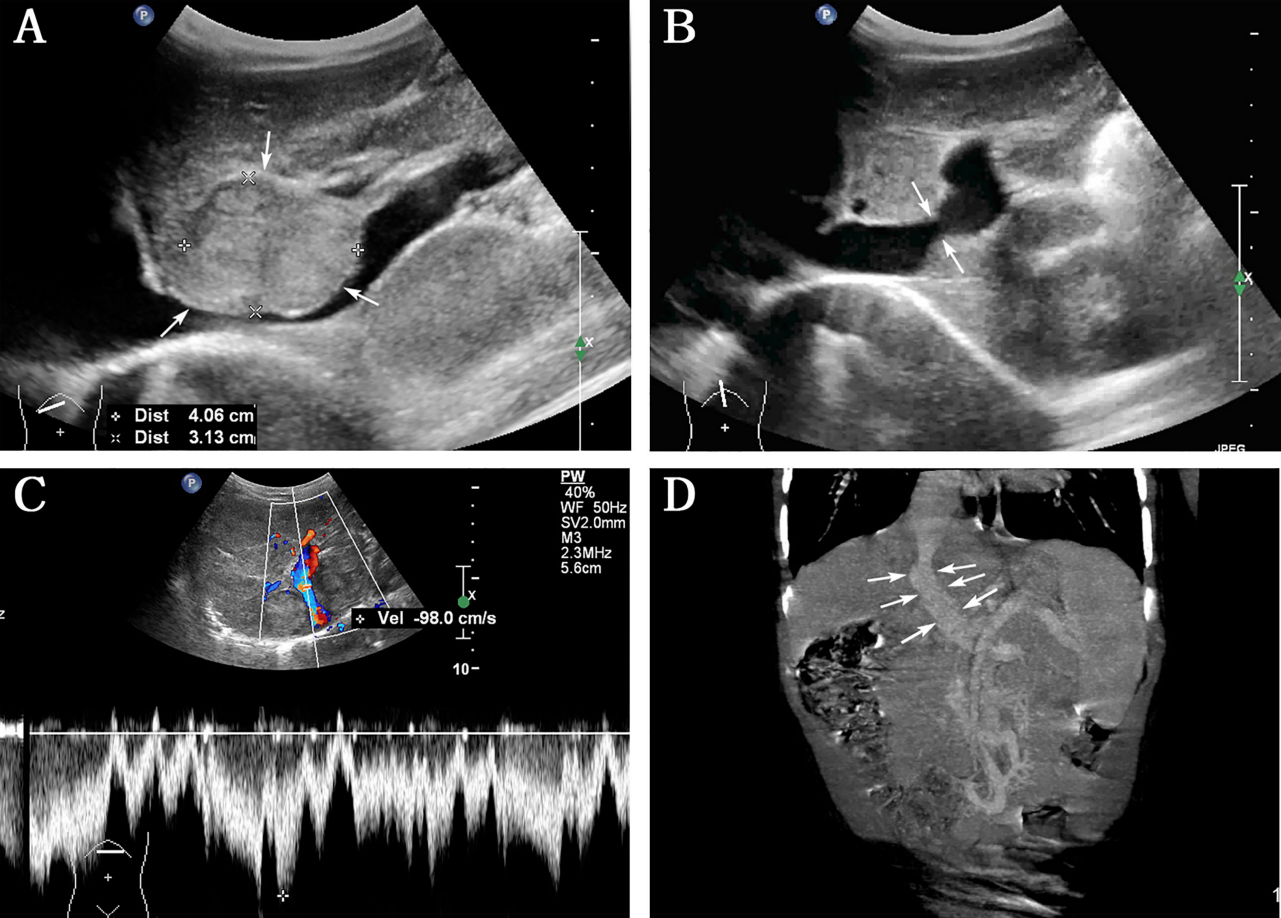
Abdominal ultrasound and contrast-enhanced computed tomography (CT) were performed before interventional therapy. **(A)** Grayscale indicates the largest hepatic nodule (arrows) with clear boundary and inferior vena cava (IVC) compression; **(B)** left portal vein directly communicated with IVC (arrows); **(C)** color Doppler ultrasound image confirmed PV flow toward IVC, with a flow velocity of 98 cm/s in the shunt; **(D)** contrast-enhanced CT showed the presence of direct communication between PV and IVC (arrows).

Abdominal US examination revealed multiple hepatic nodules with a maximum size of 4.0 × 3.1 cm (**Figure 1A**), clear boundary, and non-uniform internal echo, leading to local inferior vena cava (IVC) compression. The following notable findings were reported: the left PV (LPV) directly communicated with IVC (1.2 cm in diameter); color Doppler US confirmed the PV flow toward IVC (**Figure 1B**), the flow velocity in the shunt was 98 cm/s (**Figure 1C**), the right PV (RPV) was not shown, and no abnormal echo was observed in IVC; and splenomegaly (2 cm below the ribs). The rest of the abdominal organs showed no abnormal findings, and the abdominal cavity had no effusion. The patient was believed to have ectopic PV drainage (PV communicating with IVC), with unknown nature of multiple nodules. We recommended that the patient should undergo an additional abdominal contrast-enhanced computed tomography (CT) and digital subtraction angiography (DSA) to better understand this anatomic anomaly and clarify the nature of the nodule.

Laboratory tests showed the total bilirubin (TB; 40.6 μmol/L; reference range: 1.7–25.0 μmol/L), direct bilirubin (DB; 13.2 μmol/L; reference range: 0.0–7.1 μmol/L), indirect bilirubin (27.4 μmol/L; reference range: 1.7–19.0 μmol/L), alanine aminotransferase (ALT) (68 U/L; reference range: 3–50 U/L), aspartate aminotransferase (59 U/L; reference range 8–40 U/L), alkaline phosphatase (296 U/L; reference range: 20–145 U/L), and glutamyl transpeptidase (233 U/L; reference range: 3–40 U/L) levels. Prothrombin time (PT) extended to 28.5 s (reference range: 12.0–14.8 s) and the prothrombin activity (PTA) was 28.0% (reference range: 80.0–120.0%). Furthermore, the blood ammonia level increased to 80 µmol/L (reference range: 10.0–47.0 µmol/L) without any evidence of HE.

Based on the abovementioned findings and personal preference, he was admitted to Children’s Hospital, Zhejiang University School of Medicine to perform contrast-enhanced CT, which confirmed the presence of direct communication between PV and IVC (**Figure 1D**). Contrast-enhanced CT revealed the presence of hepatic nodules, which were misinterpreted as hemangiomas.

Subsequently, he was hospitalized in several hospitals in Shanghai, where lung CT, electrocardiogram, tumor marker (alpha fetoprotein, carcinoembryonic antigen, and carbohydrate antigen 199) levels, hepatitis B antigen–antibody, and cranial magnetic resonance imaging (MRI) showed normal results. Hepatic nodule puncture was performed (three puncture tissues at the center and edge of hepatic nodules were collected), with a pathological diagnosis of focal nodular hyperplasia (FNH) or nodular regenerative hyperplasia (NRH). DSA revealed hepatic vascular malformation, LPV was closely associated with IVC, and RPV was not clearly visualized. He was finally diagnosed with intrahepatic portosystemic shunts (IPSS), and interventional therapy was used to occlude the shunt.

**Figure 2:**
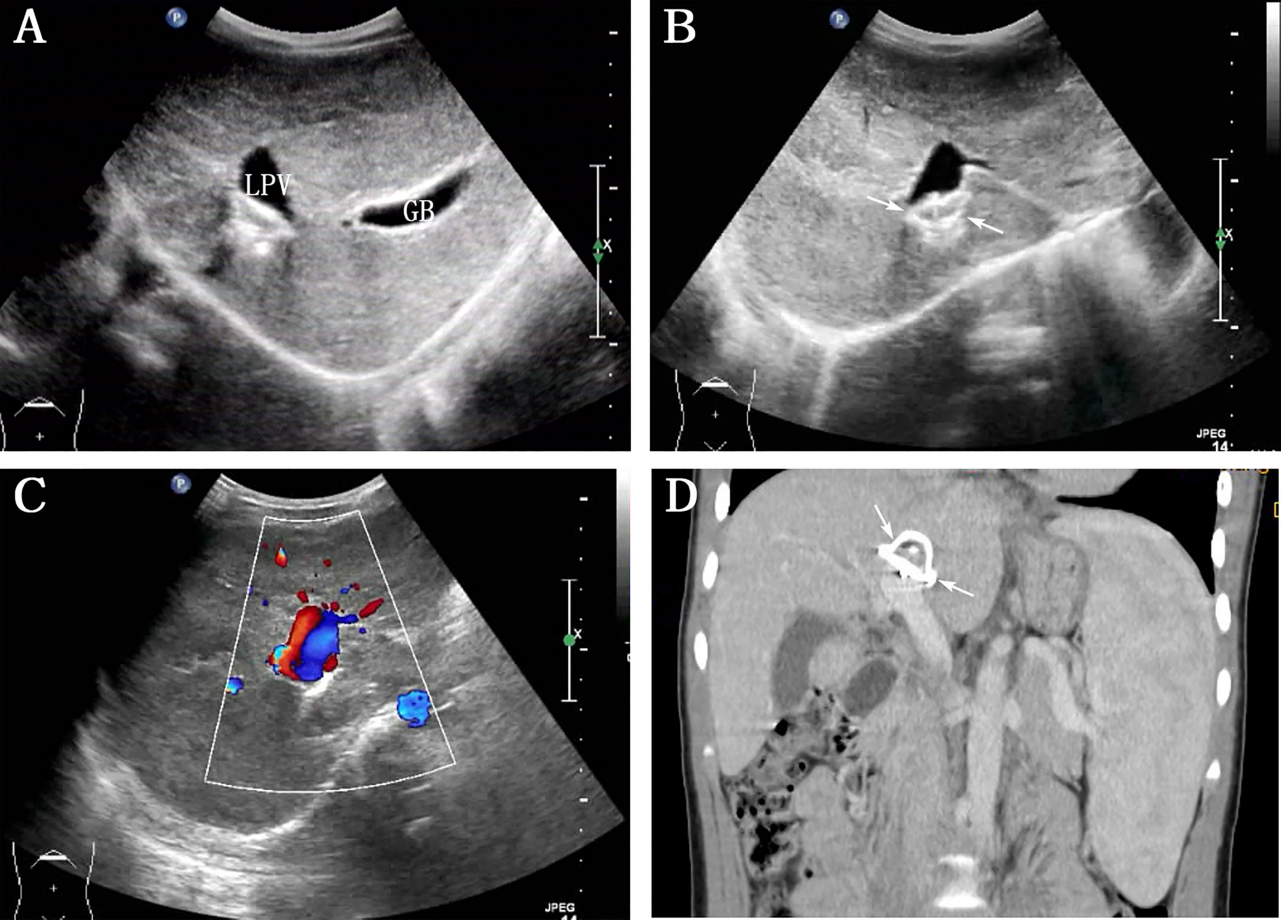
Abdominal ultrasound and contrast-enhanced computed tomography (CT) reexamination were performed 3 years after the interventional therapy. **(A)** Grayscale indicates the complete disappearance of multiple intrahepatic nodulars (LPV: left portal vein, GB: gall bladder); **(B)** hyperechogenicity between LPV and inferior vena cava (arrows); **(C)** color Doppler ultrasound image showed bidirectional venous waves in the intraportal spectrum; **(D)** contrast-enhanced CT on this reconstructed coronal view showed annular metal density shadows in the hilum of the liver (arrows).

**Figure 3:**
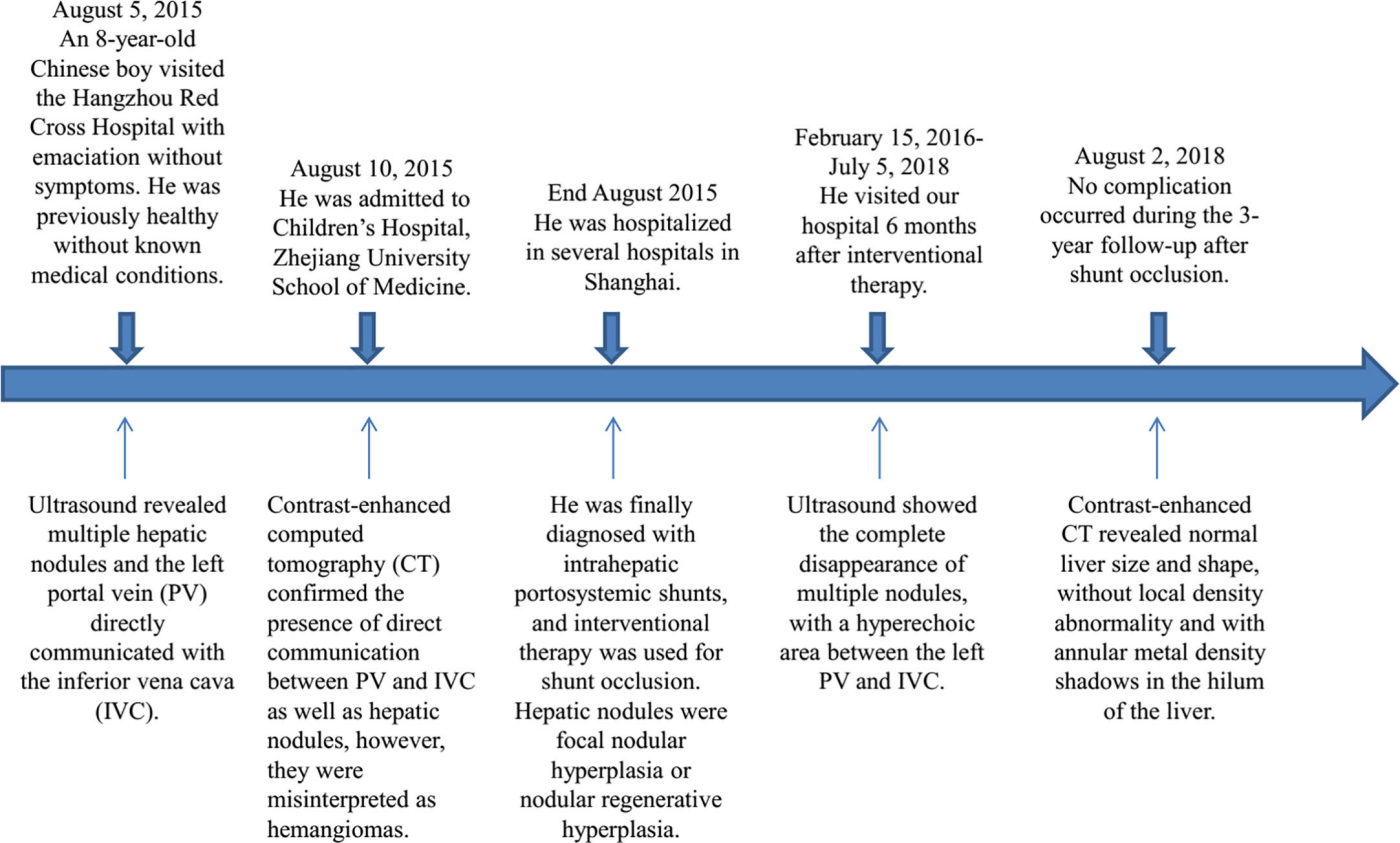
The flowchart from disease discovery to patient diagnosis and treatment.

The patient visited our hospital 6 months after the shunt closure. Abdominal US revealed the complete disappearance of multiple FNH/NRH (**Figure 2A**), and the diameter of LPV was 1.3 cm, with a hyperechoic area of 2.1 × 1.3 cm between LPV and IVC (**Figure 2B**). Subsequent CT examination confirmed that this hyperechoic area was a metal occluder placed after the interventional therapy. Color Doppler US showed bidirectional venous waves in the intraportal spectrum (**Figure 2C**). Blood tests were repeated several times after the operation, which showed normal blood ammonia level and liver function gradually. No complication occurred during the 3-year follow-up after the shunt occlusion. Abdominal contrast-enhanced CT revealed normal liver size and shape, without local density abnormality and with annular metal density shadows in the hilum of the liver 3 years after the interventional therapy (**Figure 2D**). The process of disease discovery to patient diagnosis and treatment is shown in **Figure 3** as a flowchart.

## 3 Discussion

CPSS is a malformation wherein the splanchnic vein is directly shunted into the systemic circulation *via* abnormal vascular connections, and is categorized into extrahepatic portosystemic shunts (EPSS, also known as “Abernethy malformations”) and IPSS (between PV branches and the hepatic veins or IVC) (4, 6). IPSS can be further classified into five types: type 1 is a connection between the main PV branch and IVC; type 2 is a peripheral vascular connection confined to a single hepatic segment; type 3 is a connection through a PV varix; type 4 is diffuse small connections; and type 5 involves the ductus venosus (2, 7). The current case was type 1 IPSS (LPV–IVC). Prematurity may be a risk factor for IPSS; however, its characteristics and natural history are unclear (8).

In 1956, IPSS was first reported by Doehner et al. (9). In 2000, the reported number of IPSS cases was fewer than 50 (10). Recently, however, the number of reported cases has been increasing with improvements in imaging techniques and enhanced IPSS recognition. Cytter-Kuint et al. (8) reported that IPSS without other hepatic abnormalities was a benign, self-limiting condition and in 15 infant cases, all shunts closed spontaneously without major sequelae. IPSS has a smaller size and reduced blood flow compared with EPSS; hence, it is expected to have fewer complications and a better chance of spontaneous closure. Studies have shown a spontaneous closure ratio of approximately 47% in IPSS compared with 4% in EPSS (3). However, the spontaneous closure had no established predictors. In our patient, IPSS did not exhibit spontaneous closure, which may be related to the presence of hepatic nodules and the size of the shunt. It has been reported that small intrahepatic shunts may spontaneously disappear by the age of 1–2 years, but the large shunts may persist throughout life and carry the risks of complications (4, 11). Sokollik et al. (12) recommended shunt closure in cases where severe complications occurred or IPSS did not disappear even at around 2 years of age.

The methods of closing the shunt include interventional therapy (endovascular), surgical approach and liver transplantation. Interventional therapy is the treatment of choice for most IPSS (1–3). This case was treated with interventional therapy, Multiple hepatic tumors disappeared following 6 months, which is consistent with case C reported by Albers. A 5-year-old boy’s hepatic nodules were also misinterpreted as hemangiomas by MRI. Finally, repeat US and re-review of previous imaging confirmed the presence of CPSS between the main PV and the IVC. Hepatic nodule histology was NRH/FNH. In addition, pulmonary hypertension. The difference was that he used surgical approach to close the shunt, and later on the disappearance of hepatic nodules, the pulmonary hypertension disappeared after 2 years after closure (2). The indication for another case to choose surgical shunt closure was that the FNH increased from 2 cm to 8 cm in 8 years follow-up: a failed attempt at interventional closure. Almost complete regression of the FNH after the surger (13). The evolution of liver tumor is consistent with the results reported by previously, stating that benign nodules (even for large nodules) resulting from IPSS show a good prognosis (4, 5). Tyraskis et al. (14) tracked the tumor evolution of 11 benign tumors after shunt closure. Of these, partial (4 patients) or complete (3 patients) tumor regression was observed in 7/11 (64%) patients at shunt closure, 3 patients had no change, and 1 patient presented with malignant transformation 6 months after shunt closure. In our case, the liver tumor completely disappeared after the shunt was closed, which may be related to the cause of tumor formation.

CPSS could develop various clinical features due to different pathophysiologies (15) associated with multisystem complications, with the most common being liver tumors and being present in a wide range of benign (NRH, FNH, adenomas, and hemangiomas) and malignant (hepatoblastomas, hepatocellular carcinomas, and sarcomas) hepatic nodules (14). Of these, the most common are FNH and NRH (6). It is well known that approximately 70% of the liver transfuses blood from the portal venous system during the postnatal period; however, the presence of IPSS reduces portal flow. It has been reported that the absence of intrahepatic portal flow has been known to lead to a significantly higher rate of hepatic tumor formation (16). Thus, in our case, IPSS may alter the growth factor and sex hormone metabolism owing to reduced portal venous flow and compensatory increases in hepatic arterial flow, which could drive FNH formation (14). FNH is a non-neoplastic lesion characterized by benign appearing hepatocytes with vascular anomalies and ductal proliferation (13). As reported in a previous study, the main cause of FNH is abnormal blood perfusion, which could involve either PV or arteries (17). In our case was followed-up for 6 months to 3 years, and no new liver tumor was found, indicating that shunt is closed, portal flow contributes to liver health and growth. Contrast-enhanced CT revealed FNH with a typical central stellate scar and spoke-like vascular structures (18). However, the characteristic features in our case were not obvious and were misinterpreted as hemangiomas.

CPSS identification and continued surveillance are essential, especially since timely intervention can improve clinical outcomes in the appropriate circumstances (6). The diagnosis of CPSS relies on imaging and is mostly diagnosed using DSA, which can identify the presence, location, type of CPSS, as well as PV circulation patency (10, 15). Furthermore, the development of prenatal imaging has enabled prenatal diagnosis, which has led up to 42% of CPSS cases, particularly IPSS cases, to be currently diagnosed prenatally (19). Color Doppler US can detect CPSS *via* direct shunt visualization or indirect signs, such as the diameter of the involved vessels (PV and hepatic veins), diameter of PV at the afferent side of the fistula, and size of the liver and spleen (8, 19); however, prenatal CPSS diagnosis requires US reexamination after birth due to changes in circulation. Moreover, color Doppler US is extremely useful in the follow-up of CPSS after treatment (5). In our case, no complications were found during the US follow-up for 3 years following shunt closure; therefore, it is recommended as a screening and monitoring tool (3). However, US has a few limitations: first, the surveillance and imaging protocols were not uniform. Second, the detection of the shunt may not be accurate due to several factors, such as gastrointestinal gas interference, atrophic liver, and limited imaging characteristics. Third, it is often not possible to clearly distinguish EPSS from IPSS. Fourth, EPSS away from the liver may be difficult to detect by US. Xu et al. (15) reported that two IPSSs cases and one EPSS case were missed by abdominal US and later detected by contrast-enhanced CT or MRI. Therefore, contrast-enhanced CT or MRI should be performed to further evaluate the shunt and observe other accompanying abnormalities when clinicians suspect CPSS in patients with negative US results. In summary, each type of examination could provide and supplement the relevant CPSS information.

Studies showed hyperammonemia in 79% (123/156) of children with CPSS and abnormal liver function in 78% (42/54) (4, 13), possibly because venous blood from the intestines and spleen bypasses the liver and directly diverts into the systemic circulation through the abnormal vessels (14). These values decrease after the portal flow restoration and return to normal after several days. Both are useful markers for monitoring the effectiveness of shunt closure (4, 19). Further, the presence of shunts can impair hepatic synthesis, resulting in coagulation disorders (14). PT was prolonged in 40% (31/77) of children involved in a study by Bernard et al. (4). Our patient showed increased blood ammonia levels, abnormal liver function, and prolonged PT at the hospital visit, which did not reach the degree of cholestasis and HE, and the laboratory parameters gradually returned to normal over time following shunt closure, suggesting that interventional therapy was effective for the boy.

In summary, CPSS may be asymptomatic or present with multisystem diseases of varying severity, mimicking common or rare pediatric conditions. Although CPSS is less common, it has gradually been acknowledged by clinicians, which is important for early diagnosis considering the potentially serious clinical consequences. Color Doppler US imaging is free of ionizing radiation and well tolerated, and hence, has become the first choice for assessing the pediatric abdomen. Therefore, color Doppler US is recommended as a noninvasive imaging technique to identify CPSS, monitor the therapeutic response, and conduct follow-up to guide the management of this disease and improve its outcomes.

## Data availability statement

The raw data supporting the conclusions of this article will be made available by the authors, without undue reservation.

## Ethics statement

The studies involving human participants were reviewed and approved by Hangzhou Red Cross Hospital. Written informed consent to participate in this study was provided by the participants’ legal guardian/next of kin. Written informed consent was obtained from the minor(s)’ legal guardian/next of kin for the publication of any potentially identifiable images or data included in this article.

## Author contributions

YZ: manuscript writing. TY: data collection. YM: data collection. WZ: data collection. YZ: data collection. GY: supervision and data collection. All authorscontributed to the article and approved the submitted version.
